# Late Postoperative Hemorrhage in the Gastric Remnant Following Roux-en-Y Gastric Bypass: A Case Report

**DOI:** 10.7759/cureus.85245

**Published:** 2025-06-02

**Authors:** Joshua J Yoon, Thuyduong Nguyen, Brandon Raquet, Chetan Pai

**Affiliations:** 1 Internal Medicine, Edward Via College of Osteopathic Medicine, Blacksburg, USA; 2 Gastroenterology, Mary Washington Healthcare, Fredericksburg, USA

**Keywords:** bariatric surgery complications, gastric ulcer perforation, gastrointestinal hemorrhage/etiology, roux-en-y gastric bypass, upper gastrointestinal bleed

## Abstract

The Roux-en-Y gastric bypass (RYGB) is a commonly performed bariatric surgery option for individuals with severe obesity. Although gastrointestinal (GI) bleeds are a well-recognized complication of RYGB, it is uncommon in the late postoperative period as the surgical sites heal. It can be challenging to diagnose and manage these GI bleeds due to their isolated location and, consequently, difficult accessibility via traditional endoscopy. Various approaches have been described for the management of this complication with different treatment options, and treatment can vary based on patient risk factors and presentation. We report a case of a patient with bleeding in the gastric remnant 18 years after bypass surgery. Traditional endoscopy and other imaging options were attempted before surgical intervention, with laparoscopy and intraoperative endoscopy being warranted. The patient was discharged in stable condition after spontaneous hemostasis following a complicated hospital course.

## Introduction

Obesity is a growing epidemic in the United States, as nearly 40% of adults were estimated to be obese in 2016, and this number is projected to rise to 50% by 2030 [1]. The rapidly increasing rate of obesity has led to the development of multiple treatment options adjunctive to patient education and lifestyle changes, including medications and surgical treatment. The Roux-en-Y gastric bypass (RYGB) is a common bariatric surgery option that can provide sustainable long-term weight reduction and improve management of comorbid conditions, including type 2 diabetes, hypertension, and obstructive sleep apnea [2].

Gastrointestinal (GI) bleeding is an uncommon potential complication that can occur after RYGB and is even more uncommon during the late operative period [3]. Bleeding at the bypassed portion of the stomach can further complicate diagnosis and management due to the altered anatomy and often requires careful investigation, including endoscopy, CT imaging, or surgical re-exploration. If not detected and managed in time, GI bleeds can become a major source of morbidity and mortality [3]. We present a case of GI bleeding that occurred within the gastric remnant of a patient who had undergone RYGB 18 years prior.

## Case presentation

A 73-year-old male with a history of type 2 diabetes mellitus, hyperlipidemia, and hypertension and an RYGB that had been performed 18 years prior, presented to the emergency department for evaluation of a fall and brief loss of consciousness that occurred earlier that afternoon. Notably, he mentioned several episodes of melenic stool in the past two weeks. The patient reported that he was walking down the stairs when he began to feel lightheaded and subsequently fell to the ground after a brief loss of consciousness. On examination, he was hypotensive (73/61 mmHg) and had tachycardia (114 bpm). A CT of the head and multiple plain radiographs were ordered without any significant findings. His hematologic labs revealed anemia with a hemoglobin of 7.2 g/dL and hematocrit of 21%. Given his hemodynamic status, the patient was admitted and given multiple boluses of intravenous fluids, and gastroenterology was consulted to rule out GI bleeding due to his history of melena and current anemia. An esophagogastroduodenoscopy (EGD) was performed to rule out a GI bleed, and was negative for ulcers and active bleeding and revealed a healthy appearing Roux-en-Y bypass with well-healed surgical anastomotic sites.

Following the EGD, the patient was initially managed conservatively and received two units of packed red blood cells (pRBC) and pantoprazole. However, the patient's hemoglobin dropped to 6.9 g/dL on hospital day three, so two more units of pRBC were transfused. With the patient’s hemodynamic status failing to stabilize after multiple transfusions, gastroenterology was reconsulted for repeat EGD with endoscopic ultrasonography (EUS) and possible colonoscopy. The abdominal CT revealed distension of the excluded portion of the stomach with a massive lesion occupying the gastric remnant. A repeat EGD showed no acute changes or signs of active bleeding. The EUS found a large heterogeneous mass occupying a large portion of the surgical stomach, which was then biopsied via fine-needle aspiration (FNA). Grossly, the samples appeared to be a heterogeneous mixture of gelatinous blood tissue, likely from coagulated blood products, and were sent to cytology for further review.

**Figure 1:**
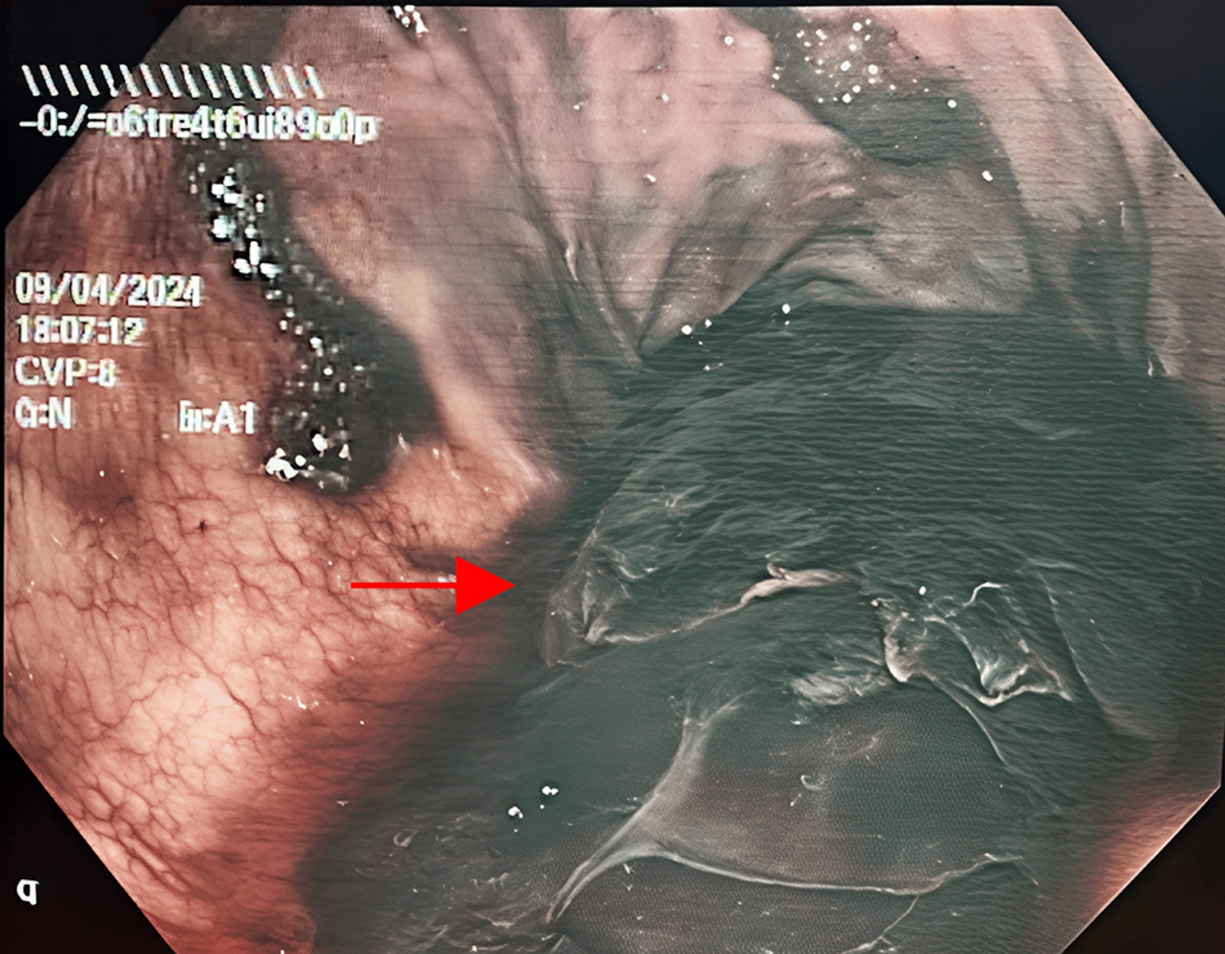
Intraoperative endoscopic view of the gastric remnant showing coagulated blood without active hemorrhage (arrow).

With more clues pointing towards anemia due to bleeding at the gastric remnant, general surgery was consulted for possible surgical management. To evaluate for any residual bleeding, a computed tomography angiography (CTA) was also performed, but no site of active GI bleeding could be identified. Despite normal imaging and transfusion of six units of pRBCs, the patient’s hemoglobin continued to drop. A decision was then made to proceed with a diagnostic laparoscopy with intraoperative endoscopy through percutaneous gastrostomy (Figure 1).

Endoscopic view of the gastric remnant revealed a large amount of coagulated blood without active bleeding at the time. Retrieval of the large coagulated mass was attempted using a Roth Net (Dublin, Ireland: STERIS), but was mostly unsuccessful due to its gelatinous consistency. The area was then extensively irrigated and suctioned to remove as much of the blood as possible. No further intervention was performed, as there seemed to be no signs of active bleeding. A gastrostomy tube was placed for drainage of any remaining fluids, and the patient was closely monitored postoperatively.

Throughout the next several days, the patient was able to maintain a stable hemoglobin and hematocrit without the need for additional blood transfusions. However, there was still a concern for recurrent bleeding; thus, bariatric surgery was consulted for consideration of definitive management by partial or total resection of the gastric remnant. After careful consideration by the bariatric surgery team, it was determined that revisional surgery would not be warranted at this time since the patient had achieved spontaneous hemostasis without the need for transfusion. The patient was observed for several more days before being discharged in stable condition with high-dose pantoprazole for long-term prevention of future bleeding episodes.

## Discussion

Gastrointestinal bleeding is a rare but concerning complication of RYGB, and can be categorized into early (<30 days postoperatively) and late (>30 days postoperatively). Early bleeding has an incidence rate of 2.6-3.1% and is usually caused by failure of the surgical anastomosis, inadequate healing at the staple site, or ulcers [3]. There are several case reports describing late bleeding, but it remains exceedingly uncommon, such that an incidence rate cannot yet be accurately quantified. These rare cases of bleeding in the late postoperative period have been described to be caused by marginal ulcerations or erosions. Ulcers that are readily accessible by endoscopy can be treated routinely in a manner similar to typical upper GI ulcers.

However, ulcers at the bypassed portion of the stomach can be much more difficult to manage. Risk factors for ulceration of the gastric remnant are similar to peptic ulcer disease, including *Helicobacter pylori* colonization, smoking, alcohol usage, and history of nonsteroidal anti-inflammatory drug (NSAID) usage [4]. The altered anatomy in the setting of RYGB proves challenging for endoscopic access and alternatives to the traditional EGD should be considered. Surgical approaches can be employed to create a percutaneous gastrostomy at the excluded stomach for introduction of the endoscope and direct hemostasis. Endoscopy can also be done intraoperatively during explorative laparotomy if standard management proves difficult [5].

Early localization of the bleeding source in patients with suspected GI hemorrhage is critical for reducing morbidity, especially in the setting of those who have undergone RYGB. In cases with negative endoscopy results, bleeding within the gastric remnant, biliopancreatic limb, or distal duodenal ulcers should be considered. Other diagnostic tools that may be useful for further evaluation following uneventful endoscopy include CTA, tagged red blood cell scan with technetium-99m, and celiac angiography [6]. Patients' hemoglobin and hematocrit should be carefully monitored and appropriately transfused to maintain hemodynamic status. Further deterioration of the patient's hemodynamic status may warrant further evaluation by surgical exploration.

Clinical manifestations may also provide valuable clues for locating the source of hemorrhage. Patients presenting with melena usually are hemorrhaging from the excluded stomach, while hematochezia often signifies bleeding at the biliopancreatic limb [3]. This is congruent with the present case, as this patient’s melena originated from bleeding within the excluded stomach.

Endoscopic access into the excluded stomach is extremely challenging, even with pediatric endoscopes, as the biliopancreatic limb can exceed 100 cm [6]. Definitive management of gastrointestinal bleeding within the gastric remnant often will require surgical intervention, and is often complicated by high morbidity and mortality in emergent settings [7]. Adjunctive pharmaceutical management is also necessary, as the gastric remnant often continues to secrete gastrin after RYGB; thus, patients are often prescribed lifelong proton pump inhibitor (PPI) medication to reduce the occurrence of peptic ulcers [8]. Spontaneous hemostasis may be achieved, as in the present case; however, definitive management by resection of the bleeding gastric remnant can be considered if there is an increased risk of recurrent bleeding.

## Conclusions

As clinicians continue to manage obesity within the United States, bariatric surgery and its complications will continue to be of clinical significance to many physicians. Gastrointestinal bleeding at the excluded stomach is a rare complication for patients with RYGB, and can be difficult to diagnose and manage. Although endoscopy is often the gold standard for both diagnosis and management of typical upper gastrointestinal bleeding, its use is often limited in ruling out typical upper gastrointestinal hemorrhage patterns in patients with gastric remnant hemorrhage. Extensive diagnostic radiographs can be indeterminate, and it is critical to not delay surgical intervention in patients with persistent hemodynamic instability. A more complete understanding of the altered anatomy in RYGB, as well as awareness of complications occurring in the late postoperative period, can help reduce morbidity and improve patient care.
